# The Biogeochemical Role of Baleen Whales and Krill in Southern Ocean Nutrient Cycling

**DOI:** 10.1371/journal.pone.0114067

**Published:** 2014-12-03

**Authors:** Lavenia Ratnarajah, Andrew R. Bowie, Delphine Lannuzel, Klaus M. Meiners, Stephen Nicol

**Affiliations:** 1 Institute for Marine and Antarctic Studies, University of Tasmania, Hobart, Tasmania, Australia; 2 Antarctic Climate and Ecosystems Cooperative Research Centre, University of Tasmania, Hobart, Tasmania, Australia; 3 Australian Antarctic Division, Kingston, Tasmania, Australia; University of Shiga Prefecture, Japan

## Abstract

The availability of micronutrients is a key factor that affects primary productivity in High Nutrient Low Chlorophyll (HNLC) regions of the Southern Ocean. Nutrient supply is governed by a range of physical, chemical and biological processes, and there are significant feedbacks within the ecosystem. It has been suggested that baleen whales form a crucial part of biogeochemical cycling processes through the consumption of nutrient-rich krill and subsequent defecation, but data on their contribution are scarce. We analysed the concentration of iron, cadmium, manganese, cobalt, copper, zinc, phosphorus and carbon in baleen whale faeces and muscle, and krill tissue using inductively coupled plasma mass spectrometry. Metal concentrations in krill tissue were between 20 thousand and 4.8 million times higher than typical Southern Ocean HNLC seawater concentrations, while whale faecal matter was between 276 thousand and 10 million times higher. These findings suggest that krill act as a mechanism for concentrating and retaining elements in the surface layer, which are subsequently released back into the ocean, once eaten by whales, through defecation. Trace metal to carbon ratios were also higher in whale faeces compared to whale muscle indicating that whales are concentrating carbon and actively defecating trace elements. Consequently, recovery of the great whales may facilitate the recycling of nutrients via defecation, which may affect productivity in HNLC areas.

## Introduction

Large regions of the Southern Ocean are characterized by low phytoplankton biomass despite high concentrations of major nutrients (e.g. nitrate, phosphate and silicate), and have been characterised as High Nutrient Low Chlorophyll (HNLC) waters [1]. Phytoplankton forms the base of the marine food chain, supporting everything from microscopic animals to large marine mammals [2]–[4]. It also plays an important role in carbon sequestration by converting carbon dioxide (CO_2_) to biomass through photosysnthesis, and through sinking, transferring the carbon to the deep ocean and sea floor sediments [5], [6]. Marine ecosystems can either act as a source or sink of atmospheric CO_2_ depending on the relative rates of photosynthesis and overall total respiration. One factor responsible for limiting the accumulation of phytoplankton in HNLC waters has been the availability of essential trace elements, particularly iron (Fe), that are required for biochemical processes such as photosynthesis and respiration, as well as in the reduction of inorganic nitrogen species [7].

The major sources of trace elements in marine ecosystems are from atmospheric deposition, continental run-off, shelf sediments, hydrothermal vents and ocean crust [8]. However the Southern Ocean is remote from most of these sources; consequently the concentration of trace elements in surface waters is low. Some of the important trace elements underpinning biogeochemical processes are: Fe and manganese (Mn) for carbon fixation; zinc (Zn), cadmium (Cd), and cobalt (Co) for CO_2_ acquisition; Zn and Cd for silica uptake by large diatoms; Co and Zn as calcifiers; Fe for nitrogen (N_2_) fixation; copper (Cu) and Fe for nitrification, denitrification and organic N utilization; Zn for organic phosphorus (P) utilization; Fe for synthesis of photopigments; and Cu for methane oxidation [9], [10]. As Fe, Mn, and Cu have a short residence time, while Cd, Zn and P have an intermediate residence time in oxygenated waters [11]–[14], any mechanism that can increase the persistence of trace elements in surface waters should enhance overall marine primary productivity.

Until recently, the primary biogeochemical role of marine animals was considered to be as consumers of carbon, converting it into fast-sinking faecal material or returning it to the atmosphere through respiration [15]. However, a number of recent studies instead suggest that marine animals and seabirds are part of a positive feedback loop that retains nutrients in the surface waters, thus enhancing primary productivity and stimulating carbon export [16]–[20].

All animals require a range of nutrients that they mostly obtain from their diet. Different marine animal groups have requirements for particular nutrients: e.g. crustaceans require Cu for their respiratory pigment [21], whereas marine mammals require Fe for the oxygen (O_2_) storage protein in muscles; myoglobin [22]. Thus animals tend to concentrate the range of nutrients that are important for their metabolic processes. Marine mammals, being air-breathing, spend most of their lives in the surface layer and are thought to defecate exclusively in the euphotic zone [23]. In addition, some animals inhabit or migrate to water deeper than the euphotic zone, where they feed and then return the scavenged nutrients to the surface layer when they defecate [24], [25]. Animals such as seabirds and whales are capable of converting the concentrated elements found in solid form in their prey into a liquid form in their faecal material that is released into the euphotic zone [16], [20], [26]. This plume of liquid, rich in trace elements, could act as a fertiliser for phytoplankton production [20], [27]. Dense aggregations of large animals may also have a significant local effect on mixing of water and nutrients across the thermocline by generating turbulence [28].

The objective of our study was to determine the degree to which a variety of trace elements are concentrated in krill tissue, and subsequently taken up into whale muscle or defecated, to evaluate their potential role in recycling nutrients in the Southern Ocean. In addition to Fe, we report the concentrations of carbon and six other biologically important elements (Cd, Mn, Co, Cu, P and Zn) measured in five species of baleen whales and four species of krill, including Antarctic krill (*Euphausia superba*). Iron concentrations and diet analysis on these samples have been presented and discussed in Nicol et al. [16] and Jarman et al. [29], respectively.

## Methods

### Sample collection

Whale muscle samples were collected from stranded and dead blue (*Baleoptera musculus*) and fin (*Baleoptera physalus*) whales in South-western Australia. Blue, fin, sperm (*Physeter macrocephalus*), humpback (*Megaptera novaeangliae*) and pygmy blue (*Baleoptera musculus brevicauda*) whale faecal samples were collected opportunistically from a range of locations by trawling 0.5 mm mesh nets over the surface waters following defecation. Four species of krill (*Nyctiphanes australis, Meganyctiphanes norvegica, Euphausia pacifica* and *Euphausia superba*) were collected from various locations worldwide. All sample tissue and faecal matter were stored in individual 50 ml polycarbonate screw cap bottles, preserved in>70% ethanol and frozen at −20°C until analyses.

### Analysis of the trace element concentration

Samples were dried at 60°C until constant weight was attained. Subsequently they were crushed using an acid-cleaned pipette tip and shaken vigorously to homogenise the samples. Digestion of 2–100 mg subsamples were performed in acid-cleaned 15 ml Teflon perfluoroalkoxy (PFA) vials (Savillex, Minnetonka, MN, USA) by adding 1 ml of concentrated nitric acid and 0.125 ml of hydrogen peroxide (all Ultrapure, Seastar Baseline, Choice Analytical). The samples were then heated at 125°C for 8 hours on Teflon coated digestion hotplate, housed in a bench-top fume hood coupled with HEPA filters to ensure clean input air (Digiprep, France). Identical procedures were applied to blanks (n = 6) and to two certified referenced materials (n = 5) (DORM-3 fish protein; National Research Council, Ottawa, Canada; and NIST 1566a oyster tissue; National Institute of Standards and Technology, Gaithersburg, Maryland, USA). Certified materials, blanks and samples were resuspended in 10–100 mL of 10% v:v nitric acid (Ultrapure, Seastar Baseline) and analysed by sector field inductively coupled plasma mass spectrometry (SF-ICP-MS) (Finnigan MAT ELEMENT 1 Bremen Germany), following methods described in Cullen and Sherrell [30] and Townsend [31].

### Analysis of carbon

All glass- and metal-ware in contact with the carbon samples were pre-combusted at 450°C for 12 hours. Subsamples (2–100 mg) of dried faecal matter were placed in 13 mm diameter silver capsules (Sercon, Australia) and carbon content was then determined at the Central Science Laboratory, University of Tasmania, using a Thermo Finnigan EA 1112 Series Flash Elemental Analyser (estimated precision ∼1%).

## Results

### Element distribution

Results for certified reference materials are presented in Table 1 and were found fit for purpose. Mean and standard deviation of C, Fe, Cd, P, Co, Mn, Cu and Zn for five species of whale faeces, two species of whale muscle and four species of krill are summarised in Table 2; with published comparative values of dissolved and particulate trace elements in Southern Ocean surface waters in Table 3, marine phytoplankton in Table 4, and Antarctic krill and marine mammals in Table 5. Concentrations of metals varied between the specimens. In krill tissue, the highest concentration was observed for Zn followed by Fe and Cu. In whale muscle, the highest concentration was observed for Fe followed by Zn and Cu. Lastly, in whale faeces, the highest concentration was observed for Zn, followed by Cu and Fe. Consistently, the three elements with the lowest mean concentrations in krill tissue and whale muscle and faeces were Mn followed by Cd and Co. There are some differences in concentration of the various elements between our results and published data (Table 5). These differences may be a result of seasonal or regional effects and variability in trace element concentrations in krill and baleen whales, which is a topic for future studies.

**Table 1:** Elemental analysis using sector field inductively coupled plasma mass spectrometry (SF-ICP-MS) for certified referenced material of fish protein (Certified Reference Material number: DORM-3) and oyster tissue (National Institute of Standards and Technology (NIST), Certified Reference Material number 1566a).

	Fe	Cd	P	Mn	Co	Cu	Zn
DORM-3 referenced values (mg kg^−1^)	347.00	0.29	n/a	4.6	n/a	15.5	51.3
Measured average (mg kg^−1^) (n = 5)	322.09	0.28	24865.65	2.92	0.23	14.88	69.55
Standard deviation	42.02	0.03	3281.6	0.39	0.03	1.47	39.06
Recovery (%)	92.82	96.52	n/a	63.4	n/a	95.99	135.58
NIST 1566a certified values (mg kg^−1^)	539.00	4.15	n/a	12.3	0.57	66.3	830
Measured average (mg kg^−1^) (n = 5)	477.85	4.13	29853.89	11.46	0.31	62.60	837.11
Standard deviation	11.84	0.04	1100.99	0.11	0.02	0.70	7.58
Recovery (%)	88.66	99.49	n/a	93.18	53.65	94.41	100.86

Averages listed are the mean of 5 replicates. Recovery values indicate the percentage difference between measured and certified values.

n/a – No certified value given.

**Table 2:** Carbon, phosphate and trace element concentrations (mean ± standard deviation) in Antarctic krill and whales (mg kg^−1^ dry weight).

Species	Sample type	n	Fe	Cd	Co	C (x 10^4^)	P (x 10^4^)	Cu	Zn	Mn
Pygmy blue, *Baleoptera musculus brevicauda*	Faeces	7	63.34±17	7.1±2.2	0.5±0.2	17.6±2.5	8.7±2.5	312.2±98.6	607.2±66.0	16.2±9.0
Blue, *Baleoptera musculus*	Faeces	15	161.8±106.5	29.7±8.6	1±0.8	18.5±3.2	9.8±1.9	239.5±68.6	460.8 ± 187.2	33.4±10.6
	Muscle	1	58.3±17.5	0.02	0.006±0.005	5.1	0.03±0.007	1.5±0.2	41.6±4.1	0.3
Fin, *Baleoptera physalus*	Faeces	2	237.4±45.3	42.1±13.1	2.1±1.3	22.1±0.7	12.1±0.4	290.7±11.4	407.1±52.8	30.5±6.9
	Muscle	1	215.7±45.8	0.2±0.3	0.07 ± 0.03	52.8	0.6±0.02	9.2±2.7	108.2±29.2	4.5±0.3
Humpback, *Megaptera novaeangliae*	Faeces	2	118.6±30.1	4.2±3.5	0.9 ±0.8	-	2.9±2.1	74.1±5.2	1099.0±553.0	18.2±10.7
Sperm whale, *Physeter macrocephalus*	Faeces	1	756.7	575	2.2	348.2	6.9	1635.4	2663.6	96
Average among whales	Faeces		145.9 ± 135.4	34.7±88.9	0.9±0.87	19.2±4.5	8.9±3.1	292.4±238.1	621.5±432.9	27.7±16.5
	Muscle		136.9 ± 91.6	0.11±0.19	0.04±0.04	51.9±1.2	0.4±0.2	5.3±4.5	78.9±40.9	2.4 ± 2.3
Antarctic krill, *Euphausia superba*	Whole krill	5	174.3±0.5	4±0.1	0.1	54.2	3.13±0.04	98.0±0.6	275.7±0.5	17.7±0.1
Krill, *Nyctiphanes australis*	Whole krill	5	91.4±1.1	2.8	0.1	35.9	6.6±0.01	40.7±0.2	444.8±2.6	8.0±0.1
Krill, *Euphausia pacifica*	Whole krill	5	62.1±0.6	2.3	0.1	45.2	1.4±0.009	15.6±0.2	293.6±2.3	9.2±0.1
Krill, *Meganyctiphanes norvegica*	Whole krill	10	11.3±8.9	2.2±0.5	0.04±0.02	43.2±2	1.06±0.6	44.6±11.0	90.5±40.8	2.0±0.8
Average among krill	Whole krill	25	76.6±64.1	2.7±0.8	0.08±0.03	44.3±6.6	2.8±2.3	49.1±30.5	49.13± 30.5	8.4±6.1

Carbon data for humpback whales are not available.

Krill samples were homogenates of 5 animals of each species.

Iron data for all species have been discussed in Nicol [16].

**Table 3:** Summary of dissolved and particulate trace element concentrations in surface waters from the literature (nmol L^−1^).

Sampling location	Depth (m)	Size partitioning	Fe	Cd	Co	P	Cu	Zn	Mn	C	Reference
Marguerite Bay, WAP		Dissolved									Hendry [63]
Ross Sea	0–100	Dissolved		0.34–0.86			0.43–3.3	2.2–8.2	0.33–1.2		Corami [45]
Ross Sea	0.5–375	Dissolved		0.04–0.73			1.23–2.16	0.24–5.17			Fitzwater [64]
Ross Sea	0–380	Dissolved					0.5–11.6		0.01–6.6		Grotti [65]
Weddell Sea	50	Dissolved	2.01						0.34		Westerlund and Öhman [66]
Atlantic sector	40	Dissolved		0.155–0.905							Löscher [67]
Atlantic sector	40–100	Dissolved					0.95–6.66	1.7–10.8			Löscher [68]
Indian-Pacific sector	40	Dissolved		0.25–0.27			1.2–1.4	2.3–2.4			Frew [69]
Indian-Pacific sector	40	Dissolved	0.1								Bowie [44]
Southern Ocean	0–20	Dissolved	0.03	0.34	0.02		1.78	1.01	0.08		Cullen [32]
Ross Sea	0–100	Particulate		0.011–0.097			0.05–0.733	0.2–1.2	19–198		Corami [45]
Ross Sea	0.5–100	Particulate							0.01–0.17		Fitzwater [64]
Ross Sea	0–380	Particulate					0.04–1.36		0.01–3.1		Grotti [65]
Weddell Sea	50	Particulate	2.18						0.022		Westerlund and Öhman [66]
Atlantic sector	40	Particulate		0.02–0.14							Löscher [67]
Atlantic sector	40–100	Particulate					0.026–0.222				Löscher [68]
East Antarctica	0–1	Particulate		0.001–0.018			0.017–0.070	0.020–0.805	0.007–0.141	1170	Lannuzel [70]
Amundsen Sea open ocean	8–50	Particulate	0.071–0.66			16.6–44.5			8.81–39.4		
Southern Ocean	0–20	Particulate	0.26	0.34	0.04		0.38	2.91	0.44		Cullen [32]
Overall ranges		Dissolved	0.03– 2.01	0.04–0.9	0.02		0.43–6.6	0.24–10.8	0.01–6.6		
		Particulate	2.18	0.01–0.14	0.04	16.6–44.5	0.017–1.36	0.02–2.91	0.01–198	1170	

Data from Frew [69] and Bowie [44] in the Australasian-Pacific sector are from non-fertilised surface waters.

**Table 4:** Trace element concentrations (mean ± standard deviation) in cellular phytoplankton (μmol L^−1^).

Species	Algal taxa	Sampling location	Fe	Cu	Zn	Mn	C	Reference
Unknown	Diatoms (Low Fe)	Southern Ocean	45±7		982±235	28±4		Twining and Baines [47]
	Autotrophic flagellates (Low Fe)	Southern Ocean	143±15		455±74	48±10		Twining and Baines [47]
	Heterotrophic flagellates (Low Fe)	Southern Ocean	270 ± 50		1615±484	51±8		Twining and Baines [47]
	Diatoms (High Fe)	Southern Ocean	235±27		1331±350	48±8		Twining and Baines [47]
	Autotrophic flagellates (High Fe)	Southern Ocean	715±94		971±265	77±11		Twining and Baines [47]
	Heterotrophic flagellates (High Fe)	Southern Ocean	463±57		2410 ± 643	99±18		Twining and Baines [47]
*Thalassiosira pseudona*	Diatom	Sargasso sea		21.4±6.5			13.9±0.26	Annett [53]
	Diatom	Sargasso sea		56.6±5.1			12.7±0.010	Annett [53]
*Thalassiosira oceanica*	Diatom	Sargasso sea		3.43±0.27			10.2±1.1	Annett [53]
	Diatom	Sargasso sea		79.3 ± 4.8			17.0±1.2	Annett [53]
*Skeletonema menzeli*	Diatom	Sargasso sea		4.75±0.57			10.9±0.72	Annett [53]
	Diatom	Sargasso sea		33.8±11			11.1±0.97	Annett [53]

Twining and Baines [47] - Concentrations prior to Fe fertilisation are Low Fe, and following Fe fertilisation are High Fe.

Annett [53] - We used the highest and lowest Cu concentrations measured for each species of phytoplankton and its corresponding C concentration.

**Table 5:** Summary of trace element concentrations in Antarctic krill and marine mammals from the literature (mg kg^−1^).

Species	Sample type	n	Fe	Cd	Co	Cu	Zn	Mn	Reference
Antarctic krill, *Euphausia superba*	Whole	152	0.8 – 1.45	0.2 – 0.48		3.2 – 8.1	2.2 – 4.9	0.14 – 0.4	Yamamoto [35]
	Whole	-	52.2 – 64.2	0.59 – 0.78	0.064 – 0.074	69.9 – 71.2	59.6 – 66.0	3.82 – 4.2	Barbante [34]
Adelie penguin, *Pygoscelis adeliae*	Muscle	10	109 – 204	0.04 – 0.46		2.2 – 3.05	18.9 – 27.2	0.21 – 0.35	Honda [37]
	Liver	10	233 – 1670	0.99 – 8.46		3.26 – 6.06	31.9 – 73.4	1.57 – 2.9	Honda [37]
	Kidney	10	162 – 360	23.8 – 93.4		2.89 – 4.51	29.6 – 71.4	0.95 – 2.18	Honda [37]
	Whole	10	68.7 – 163	0.33 – 1.07		1.89 – 2.2	27.1 – 35.7	0.6 – 1.02	Honda [37]
Southern minke whale, *Baleoptera acutorostrata*	Muscle	37	10.5 – 67.5	0.01 – 0.2		0.42 – 0.78	6.9 – 25.7	0.6 – 0.19	Honda [37]
	Liver	37	35.2 – 4482	2.32 – 41.7		4.25 – 11.2	30.2 – 70.1	1.6 – 4.89	Honda [37]
	Kidney	37	20.2 – 114	3.5 – 85		1.87 – 3.75	23.3 – 60.1	0.61 – 1.37	Honda [37]
	Whole	37	12.3 – 149	0.1 – 0.9		0.59 – 1.1	14.6 – 50.4	0.18 – 0.4	Honda [37]
Weddell seal, *Leptonychotes weddell*	Muscle	2	237 – 267	0.01 – 0.03		0.85 – 1.02	33.7 – 39.6	0.13 – 0.14	Honda [37]
	Liver	2	389 - 940	0.96 – 1.31		15.0 – 25.8	41.7 – 47.0	1.80– 1.86	Honda [37]
	Kidney	2	159 – 618	2.89 – 9.93		5.12 – 11.0	27.4 – 30.7	0.9 – 1.12	Honda [37]
	Whole	2	141 – 229	0.05 – 0.1		1.08 – 1.36	19.7 – 20.1	0.15 – 0.2	Honda [37]
Chinstrap penguin, *Pygoscelis antarctica*	Faeces	32	-	1.23 – 3.48		128.1 - 372.4	94.5 – 354.71		Espejo [38]
Gentoo penguins, *Pygoscelis papua*	Faeces	40	-	1.23 – 3.58		73.2 -308	110.1 – 430.8		Espejo [38]
Crabeater seal, *Lobodon carcinophagus*	Muscle	27	0.3 – 0.7	0.01-0.39	0.06 – 0.13	2.7–4.3	57 – 133	0.17 – 0.34	Szefer [36]
	Liver	27	3.0 – 28.0	4.6 – 38.5	0.1 – 0.2	42–105	89 – 230	9.5 – 17.3	Szefer [36]
	Kidney	27	0.3 – 0.69	14.3 – 90	0.17 – 0.3	18.9 – 39.5	80 – 162	2.0 – 5.0	Szefer [36]
Leapord seal, *Hydrurga leptonyx*	Muscle	3	0.57 – 0.85	0.03 – 0.1	0.07 – 0.12	2.5 – 5.4	79 – 91	0.11 – 0.14	Szefer [36]
	Liver	3	2.1 – 3.64	4.0 – 8.5	0.12 – 0.16	98 – 116	145 – 221	13.9 – 15.0	Szefer [36]
	Kidney	3	0.5 – 0.81	15.7 – 35.9	0.20 – 0.23	22.5 – 43.8	102 – 147	2.1 – 4.7	Szefer [36]
	Stomach content	4	0.57 – 0.81	0.03 – 0.06	0.06 – 0.1	13.3 – 16.4	61 – 87	0.22 – 0.25	Szefer [36]
Weddell seal, *Leptonychotes weddell*	Muscle	2	0.87 – 1.42	0.01 – 0.06	0.08 – 0.12	2.1 – 3.1	104 – 133	0.24 – 0.4	Szefer [36]
	Liver	2	1.09 – 3.57	0.8 – 5.6	0.14 – 0.19	28.0 – 87.1	147 – 189	10.4 – 15.4	Szefer [36]
	Kidney	2	0.33 – 0.51	6.9 – 44.5	0.19 – 0.21	21.7 – 24.5	88 – 158	2.1 – 4.4	Szefer [36]

Trace element concentrations in marine mammals from Honda et al. (1987) are in mg/wet kg. All other trace element concentrations are in mg/dry kg.

Mean concentrations of trace elements were higher in whale faecal matter compared to whale muscle and krill tissues. When compared to published Southern Ocean seawater concentrations in HNLC waters [32], the metal content of krill tissue was between 22 thousand (for Co) and 4.8 million (for Fe) times higher than surface water concentrations, while whale faecal matter was between 276 thousand (for Co) and 9.2 million times (for Fe) times higher.

### Metal: Carbon and carbon to phosphorus ratio

**Figure 1:**
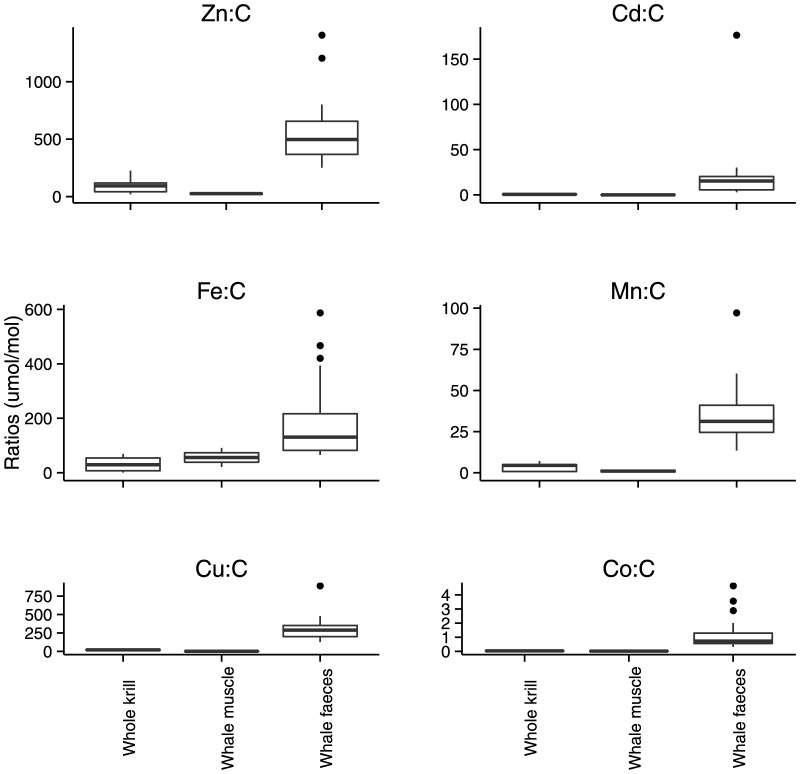
Metal to carbon ratios in krill and whales (μmol mol^−1^). Data points above the third quartile for whale faeces are 3 or more times higher than the interquartile range.

**Figure 2:**
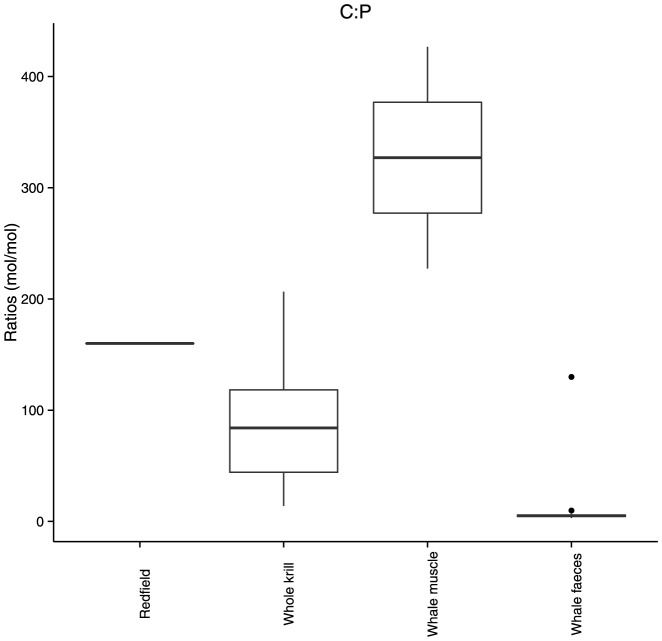
Carbon to phosphorus ratio in krill and whales (mol mol^−1^). Data point above the third quartile for whale faeces is 3 or more times higher than the interquartile range.

When normalised to C, the concentration of Cd, Cu, Co, Mn and Zn were higher in krill tissue compared to whale muscle, whereas Fe was higher in whale muscle compared to krill tissue (Table 6 and Figure 1). All metal to C ratios were higher in whale faeces compared to whale muscle. When normalised to P, the C content was highest in whale muscle followed by krill tissue and lastly whale faeces (Table 6 and Figure 2). Redfield C:P molar ratio of 106∶1 mol:mol is typical of phytoplankton [33]. Here, whale faeces and krill tissue are below the C:P Redfield ratio and whale muscle are higher.

**Table 6:** Trace metal: carbon ratios (mean ± standard deviation) in whale faeces, whale muscle and Antarctic krill (μmol mol^−1^, C:P in mol mol^−1^).

Species	Sample type	n	Fe:C	Cd:C	Co:C	Cu:C	Zn:C	Mn:C	C:P	Reference
Pygmy blue, *Baleoptera musculus brevicauda*	Faeces	7	76.5±14.3	4.3±0.98	0.5±0.2	342.7±125.7	644±120.9	19.7±9.6	5.4 ± 1	This study
Blue, *Baleoptera musculus*	Faeces	15	206.8±148.5	17.2±5.8	1.3±1.2	262±82.2	493.7±250.4	41.7±17.8	5.1±1.7	This study
	Muscle	1	21.3	0.005	0.04	3.2	14.9	0.1	426.7	This study
Fin, *Baleoptera physalus*	Faeces	2	230.2 ± 48.2	20.3±7	1.9±1.3	247.8±5.7	334.5±44.1	29.9±7.6	4.7±0.1	This study
	Muscle	1	91.04	0.009	0.04	3.2	35.6	1.9	227.3	This study
Sperm whale, *Physeter macrocephalus*	Faeces	1	467.4	176.4	1.3	887.7	1405.1	60.3	129.9	This study
Average among whales	Faeces	27	182.7±142.2	20.2±33.5	1.15 ± 1.1	308.5±154.5	559.6±281.2	35.3±18.3	10.2±25	This study
	Muscle	2	56.1±49.3	0.007±0.003	0.02 ± 0.03	1.9±1.8	25.3±14.7	1.02±1.3	327.1±141	This study
Antarctic Krill, *Euphausia superba*	Whole krill	5	69.04	0.76	0.04	34.03	93.4	7.15	44.2	This study
Krill, *Nyctiphanes australis*	Whole krill	5	54.3	0.8	0.06	21.4	226.7	4.9	13.9	This study
Krill, *Euphausia pacifica*	Whole krill	5	23.9	0.5	0.03	6.6	118.6	4.5	84	This study
Krill, *Meganyctiphanes norvegica*	Whole krill	10	4.0±4.8	0.5±0.06	0.02 ± 0.01	20.3±7.6	30.8±16.2	0.76 ±0.03	206.5	This study
Average among krill	Whole krill	25	32.1±29.5	0.6±0.2	0.03±0.01	20.5±10.4	100±81	3.6±2.8	93.4±74.6	This study
Phytoplankton	Diatoms		6	3.4	67.8					Twining and Baines [47]
	Autotrophic flagellates		8.7	2.7	22.2					Twining and Baines [47]
	Heterotrophic flagellates		14.1	3	46.9					Twining and Baines [47]
	Diatoms					0.335±0.030				Annett [53]
	Diatoms					4.46±0.40				Annett [53]

For comparison, Redfield ratio of C:P is 106∶1 mol:mol.

All data from Twining and Baines [47] are from low Fe conditions.

For data from Annett [53] we used the lowest and highest Cu:C ranges.

## Discussion

### Comparison to published analyses

The concentrations of trace elements in krill from this study were within the reported ranges for the Antarctic krill (Table 5) [34], [35]. For whale muscle, the concentration of Cd, Cu and Zn were similar to published values from other Southern Ocean marine mammals: Crabbeater seal (*Lobodon carcinophagus*), Leopard seal (*Hydrurga leptonyx*), and Weddell seal (*Leptonychotes weddellii*) (Table 5) [36].

Most studies investigating trace element concentration in marine vertebrates have used liver or kidney tissue as a means of quantifying the bioaccumulation of metal contaminants. However, as liver plays an important role in accumulation and detoxification of elements, it is expected that the concentration of elements in liver and kidney would not be comparable with trace element concentrations in muscle samples analysed in this study [37]. Unfortunately we did not have any samples from other whale tissue to compare with the literature values. The concentration of Fe, Mn, Zn, Cd and Cu in whale muscle from this study was much higher than published muscle concentrations of the Southern minke whale (*Baleoptera acutorostrata*) (Table 5) [37]. In whale faeces, the concentration of Cd, Cu and Zn were higher than published values for faeces from Antarctic chinstrap penguins (*Pygoscelis antarctica*) (Table 5) [38]. To the best of our knowledge, there are no other studies that have reported trace element concentrations in faecal matter from Antarctic vertebrates.

### Antarctic krill and baleen whales as sources of trace elements to ocean surface waters

Iron has been demonstrated to be the primary factor controlling marine primary productivity in one third of the world's oceans, including the climatically important Southern Ocean. Iron-containing proteins are essential for photosynthetic and respiratory electron transport [39], and iron been demonstrated to limit the growth rates of the diatom *Thalassiosira weissflogii* and the dinoflagellate *Prorocentrum minimum* when the unchelated Fe concentrations in seawater fall below 0.1 nmol L^−1^ [40]. This is further supported by the 100-fold increase in diatom concentrations following natural and artificial Fe-fertilization experiments in HNLC surface waters (see Boyd [41] and de Baar [42] for a synthesis).

Dissolved and particulate Fe concentration in surface seawater of HNLC regions is typically less than 1 nmol L^−1^ [32], [43], [44]. This micronutrient can be passively scavenged onto particles or actively taken up by organisms. Nicol [16] indicated that the Southern Ocean krill population could contain approximately 24% of the total Fe in the surface waters within its range, and whale faecal Fe content (145±133.7 mg kg^−1^) was approximately ten million times that of Southern Ocean surface seawater concentrations. Here we confirm that krill concentrate the Fe derived from phytoplankton into its tissue, with the Fe:C ratio in krill 3 times higher than the averaged published value for Southern Ocean phytoplankton in low Fe conditions (Table 6). In whale muscle, the Fe:C ratio was almost double that of krill and in whale faecal matter it was over 5 times higher than krill tissue. This indicates that whales are concentrating the carbon and actively defecating the Fe.

Manganese is also a crucial trace element in seawater, and it is required by the water oxidizing complex of photosystem II in phytoplankton [9]. The concentration of Mn in Southern Ocean surface waters is typically low (dissolved and particulate 0.02 – 6.77 nmol L^−1^, but 19.33 – 199.2 nmol L^−1^ in the Ross Sea [45], and 8.81 – 39.4 nmol L^−1^, particulate only, in the Amundsen Sea [46]). However published average cellular concentrations of Mn in diatoms from low Fe waters in Southern Ocean were between 200 and 2 million times higher than surface water concentrations suggesting that phytoplankton is enriched in Mn. Manganese is also an essential element for metabolism in crustaceans [48]. Accordingly, krill tissue showed even higher concentrations of Mn (8.4±6.1 mg kg^−1^), which is over 300,000 times higher than typical HNLC seawater concentrations of 0.52 nmol L^−1^ (dissolved and particulate) [32]. Whale muscle had low concentrations of Mn (2.4±2.3 mg kg^−1^), and lower Mn:C ratio compared to whale faeces. This is because Mn is not assimilated and consequently is often used as a measure of assimilation efficiency in marine mammals [49]. As a result, and similar to Fe, whales defecate most of their dietary Mn as demonstrated by high Mn content in their faeces (27.3±16.3 mg kg ^−1^) compared to their muscle (2.4±2.3 mg kg ^−1^).

The Zn, Co and Cd concentrations in Southern Ocean surface waters are low (0.24 – 9.4 nmol L^−1^,0.00006 pmol L^−1^ and 0.04 – 0.905 nmol L^−1^, respectively – Table 2), however, these elements are essential cofactors in metalloenzymes in marine phytoplankton. All marine phytoplankton have adapted to limitations of CO_2_ diffusion in water by evolving carbon concentrating mechanisms (CCMs) to support photosynthetic carbon fixation [50]. The CCM catalyses the equilibrium between bicarbonate (HCO_3_^−^) and CO_2_ using the Zn metalloenzyme carbonic anyhydrase [10]. Under Zn limitation, the carbonic anhydrase can function with Co or Cd instead of Zn [51]. Therefore the ability of marine phytoplankton to acquire CO_2_ also depends on the availability of Zn, Co and Cu in surface waters.

The mean cellular concentrations of Zn in diatoms vary by 2 orders of magnitude (3.43 – 982 µmol L^−1^ – Table 3); however diatoms show cellular accumulation of Zn, with concentrations between 1000 to 100,000 times higher than seawater (Table 4). Zinc is then further concentrated in krill tissue (275.4±137.2 mg kg^−1^). Whale muscle was relatively low in Zn (74.9±40.9 mg kg^−1^) compared to krill tissue, and Zn:C ratios were lower in whale muscle compared to whale faeces suggesting the low requirement of whales on this element. As such, most of the Zn is released through whale faecal matter (621.5±432.9 mg kg^−1^).

Cobalt and Cd were present in very low concentrations in krill tissue (0.08±0.03 mg kg^−1^ and 2.8±0.7 mg kg^−1^, respectively) suggesting that relative to other trace elements measured in this study, krill may have little use for Co and Cd. When normalised to C, Co and Cd were higher in phytoplankton compared to the average among krill (Table 6). Similarly Co and Cd were scarce in whale muscle (0.04±0.04 mg kg^−1^ and 0.1±0.2 mg kg^−1^, respectively). When normalised to C, Co and Cd were lower in whale muscle compared to whale faeces, indicating that these elements are expelled through their faecal matter (0.94±0.87 mg kg^−1^ and 34.7±88.9 mg kg^−1^, respectively). Interestingly, the concentration of Cd in sperm whale faeces was much higher compared to other species of whales in this study (575 mg kg^−1^), which may reflect the different diet of this species. Sperm whales in the Southern Ocean predominantly consume squid which may predate on Antarctic krill [52].

Copper is one element that shows clear differential uptake and utilization across the food web compared to other elements in this study. Copper concentration in seawater is low (dissolved and particulate 0.48 – 12.96 nmol L^−1^ - Table 2) and is little concentrated by phytoplankton (3.48 – 79.3 µmol L^−1^) [53], which appear to have little physiological use for it. Studies have demonstrated that Cu is toxic to the dinoflagellate *Gonyaulax tamarensis* and the diatom *T. pseudonana*, and is able to decrease their growth at only a few pmol L^−1^ [54], [55]. Krill, like most crustaceans however, require Cu, as it is an essential element in their respiratory pigment; hemocyanin [21]. Accordingly, krill tissues show a marked bio-concentration of Cu (49.1±30.5 mg kg^−1^ – Table 5, and Cu:C 20.5±10.4 µmol mol^−1^ - Table 6), 100,000 times higher than Southern Ocean surface waters and over 1.5 million times higher than that measured for Southern Ocean diatoms. Whale muscle was relatively low in Cu (5.3±4.5 mg kg^−1^) compared to their prey, which reflects the lower physiological dependency of mammals on this element. Consequently, whale faeces contained high levels of Cu (1635 5.3 mg kg^−1^ in sperm whales, 253.5±100.4 mg kg^−1^, all other species), and higher Cu:C ratio compared to whale muscle, suggesting that whales take up relatively little Cu from their diet.

Phosphorus is an essential nutrient required for structural and functional components of all organisms. Despite a high range, the mean C:P ratio in whale muscle from our study was 30 times higher than mean whale faeces ratio and 3 times higher than the Redfield ratio (Figure 2), indicating that whales are actively storing the P in their muscle. When nutrients are not limiting, the C:P ratio in most phytoplankton is 106∶1 [33]. When P is scarce, phytoplankton have been demonstrated to reduce their cellular P requirements by substituting phospholipids for non-P membrane lipids [56]. In the Southern Ocean, surface water phosphate concentrations (16.6 – 44.5 nmol L^−1^) [46] are much higher than the other elements we report here. Despite this, the concentration of P in krill was over 30 million times higher than median surface water concentrations (28,304.1±23,286.7 mg kg^−1^). Whales concentrate the P from krill for biochemical processes.

Our results suggest that Antarctic krill and whales may be a key part of marine biogeochemical cycling and act as a source of essential and limiting trace elements to phytoplankton in surface waters of the Southern Ocean. Krill and whales are long-lived, actively swimming animals that do not undergo any form of dormancy. As such, the large stock of krill can act as a mechanism of retaining trace elements in the surface waters whereas whales concentrate certain elements required for physiological processes from the krill, but actively defecate other elements that can be used for phytoplankton production. In addition, krill are capable of absorbing elements such as fluorine directly from seawater suggesting that they can concentrate some elements despite their scarcity in surface waters [57].

### Ecological importance of whales – past, present and future

The loss of large predators from marine ecosystems has the potential to affect marine biogeochemistry, and consequently marine primary productivity and carbon sequestration [16], [19], [20]. Because of their vast size and huge consumption of krill, blue and fin whales would have been the dominant krill consumers in the Southern Ocean before the era of commercial whaling and thus would have been the significant contributors to ocean nutrient recycling. Although their large size acts as a carbon store, their major role is in how they affect the recycling of critical elements, and it is the availability of these elements that affects the ocean's ability to sequester carbon. Consequently it has been suggested that the efficiency of recycling and supply of essential nutrients to surface waters has diminished in the Southern Ocean due to massive reductions in whale numbers through commercial whaling [18], [58], [59].

The pre-exploitation population of Antarctic blue whales was estimated to be between 202,000 to 311,000 individuals and was expected to have exported approximately 72,172 tons C yr^−1^ [19], [60]. Current estimates of Antarctic blue whales are approximately 4,727 individuals, less than 2% of mean pre-exploitation levels [19], [60], with a predicted recovery rate of 8.2% per year [61]. There is no reliable data on pygmy blue whale abundances. Fin whales are thought to be more abundant and their numbers may be increasing; however, current estimates of population sizes are not available. Many humpback whale populations are recovering quickly but their current numbers are still considerably below pre-exploitation population sizes. The recovery of the great whales could increase the spatial extent of productive regions in the Southern Ocean through the recycling of essential nutrients to surface layers from their faecal matter [19], [59].

## Conclusion

There is accumulating evidence of the role of whales in the ocean nutrient cycling and their importance relative to their abundance (see Nicol [16], Lavery [18], Pershing [19], Wing [20], Lavery [58], Roman [62] for synthesis). Our results show that krill can act as a reservoir of essential trace elements in surface waters, and whales can release these stored elements through feeding and defecation. This study further extends the role of larger animals as important components of ocean biogeochemical cycling for a range of elements. To fully understand the role of large marine mammals in ocean biogeochemical cycling future studies will have to determine the bioavailability of the elements contained in whale faeces, and to quantify the combined effects of, nutrient recycling in the surface layer, the effects of nutrient scavenging from deep water and biogenic turbulence caused by vertically migrating whales.
