# Correction: The Biogeochemical Role of Baleen Whales and Krill in Southern Ocean Nutrient Cycling

**DOI:** 10.1371/journal.pone.0125134

**Published:** 2015-04-17

**Authors:** Lavenia Ratnarajah, Andrew R. Bowie, Delphine Lannuzel, Klaus M. Meiners, Stephen Nicol

There is an error in Table 2. The numbers in the “Zn” column for “Average among krill” should be 255.5 ± 141.6. Please see the corrected Table 2 here.

**Table 2:** Carbon, phosphate and trace element concentrations (mean ± standard deviation) in Antarctic krill and whales (mg kg^-1^ dry weight).

**Species**	**Sample type**	**n**	**Fe**	**Cd**	**Co**	**C**(x 10^4^)	**P**(x 10^4^)	**Cu**	**Zn**	**Mn**
Pygmy blue, *Baleoptera musculus brevicauda*	Faeces	7	63.34 ± 17	7.1 ± 2.2	0.5 ± 0.2	17.6 ± 2.5	8.7 ± 2.5	312.2 ± 98.6	607.2 ± 66.0	16.2 ± 9.0
Blue, *Baleoptera musculus*	Faeces	15	161.8 ± 106.5	29.7 ± 8.6	1 ± 0.8	18.5 ± 3.2	9.8 ± 1.9	239.5 ± 68.6	460.8 ± 187.2	33.4 ± 10.6
	Muscle	1	58.3 ± 17.5	0.02	0.006 ± 0.005	5.1	0.03 ± 0.007	1.5 ± 0.2	41.6 ± 4.1	0.3
Fin, *Baleoptera physalus*	Faeces	2	237.4 ± 45.3	42.1 ± 13.1	2.1 ± 1.3	22.1 ± 0.7	12.1 ± 0.4	290.7 ± 11.4	407.1 ± 52.8	30.5 ± 6.9
	Muscle	1	215.7 ± 45.8	0.2 ± 0.3	0.07 ± 0.03	52.8	0.6 ± 0.02	9.2 ± 2.7	108.2 ± 29.2	4.5 ± 0.3
Humpback, *Megaptera novaeangliae*	Faeces	2	118.6 ± 30.1	4.2 ± 3.5	0.9 ±0.8	-	2.9 ± 2.1	74.1 ± 5.2	1099.0 ± 553.0	18.2 ± 10.7
Sperm whale, *Physeter macrocephalus*	Faeces	1	756.7	575	2.2	348.2	6.9	1635.4	2663.6	96
Average among whales	Faeces		145.9 ± 135.4	34.7 ± 88.9	0.9 ± 0.87	19.2 ± 4.5	8.9 ± 3.1	292.4 ± 238.1	621.5 ± 432.9	27.7 ± 16.5
	Muscle		136.9 ± 91.6	0.11 ± 0.19	0.04 ± 0.04	51.9 ± 1.2	0.4 ± 0.2	5.3 ± 4.5	78.9 ± 40.9	2.4 ± 2.3
Antarctic krill, *Euphausia superba*	Whole krill	5	174.3 ± 0.5	4 ± 0.1	0.1	54.2	3.13 ± 0.04	98.0 ± 0.6	275.7 ± 0.5	17.7 ± 0.1
Krill, *Nyctiphanes australis*	Whole krill	5	91.4 ± 1.1	2.8	0.1	35.9	6.6 ± 0.01	40.7 ± 0.2	444.8 ± 2.6	8.0 ± 0.1
Krill, *Euphausia pacifica*	Whole krill	5	62.1 ± 0.6	2.3	0.1	45.2	1.4 ± 0.009	15.6 ± 0.2	293.6 ± 2.3	9.2 ± 0.1
Krill, *Meganyctiphanes norvegica*	Whole krill	10	11.3 ± 8.9	2.2 ± 0.5	0.04 ± 0.02	43.2 ± 2	1.06 ± 0.6	44.6 ± 11.0	90.5 ± 40.8	2.0 ± 0.8
Average among krill	Whole krill	25	76.6 ± 64.1	2.7 ± 0.8	0.08 ± 0.03	44.3 ± 6.6	2.8 ± 2.3	49.1 ± 30.5	255.5± 141.6	8.4 ± 6.1

Carbon data for humpback whales are not available

Krill samples were homogenates of 5 animals of each species

Iron data for all species have been discussed in Nicol [16].

There is a reference missing from Table 3. Please see the corrected Table 3 here.

**Table 3:** Summary of dissolved and particulate trace element concentrations in surface waters from the literature (nmol L^-1^).

**Sampling location**	**Depth(m)**	**Size partitioning**	**Fe**	**Cd**	**Co**	**P**	**Cu**	**Zn**	**Mn**	**C**	**Reference**
Marguerite Bay, WAP		Dissolved									Hendry [63]
Ross Sea	0-100	Dissolved		0.34-0.86			0.43-3.3	2.2-8.2	0.33-1.2		Corami [45]
Ross Sea	0.5-375	Dissolved		0.04-0.73			1.23-2.16	0.24-5.17			Fitzwater [64]
Ross Sea	0-380	Dissolved					0.5-11.6		0.01-6.6		Grotti [65]
Weddell Sea	50	Dissolved	2.01						0.34		Westerlund and Öhman [66]
Atlantic sector	40	Dissolved		0.155-0.905							Löscher [67]
Atlantic sector	40-100	Dissolved					0.95-6.66	1.7-10.8			Löscher [68]
Indian-Pacific sector	40	Dissolved		0.25-0.27			1.2-1.4	2.3-2.4			Frew [69]
Indian-Pacific sector	40	Dissolved	0.1								Bowie [44]
Southern Ocean	0-20	Dissolved	0.03	0.34	0.02		1.78	1.01	0.08		Cullen [32]
Ross Sea	0-100	Particulate		0.011-0.097			0.05-0.733	0.2-1.2	19-198		Corami [45]
Ross Sea	0.5-100	Particulate							0.01-0.17		Fitzwater [64]
Ross Sea	0-380	Particulate					0.04-1.36		0.01-3.1		Grotti [65]
Weddell Sea	50	Particulate	2.18						0.022		Westerlund and Öhman [66]
Atlantic sector	40	Particulate		0.02-0.14							Löscher [67]
Atlantic sector	40-100	Particulate					0.026-0.222				Löscher [68]
East Antarctica	0-1	Particulate		0.001-0.018			0.017-0.070	0.020-0.805	0.007-0.141	1170	Lannuzel [70]
Amundsen Sea open ocean	8-50	Particulate	0.071-0.66			16.6-44.5			8.81-39.4		Planquette [46]
Southern Ocean	0-20	Particulate	0.26	0.34	0.04		0.38	2.91	0.44		Cullen [32]
Overall ranges		Dissolved	0.03– 2.01	0.04-0.9	0.02		0.43-6.6	0.24-10.8	0.01-6.6		
		Particulate	2.18	0.01-0.14	0.04	16.6-44.5	0.017-1.36	0.02-2.91	0.01-198	1170	

Data from Frew [69] and Bowie [44] in the Australasian-Pacific sector are from non-fertilised surface waters
